# 

**Published:** 1867-11

**Authors:** 


### Books and Pamphlets Received.

Catalogue of tne Surgical Section of the United States Army, Medical Museum, prepared under the direction of the Surgeon-General, U. S. Army. By Alfred A. Woodhull, Assistant Surgeon and Brevt. Major U. S. Army.

Catalogue of the Medical Section of the United States Army, Medical Museum, prepared under the direction of the Surgeon-General U. S. Army. By Brevt. Lieut. Colonel J. J. Woodward, Assistant Surgeon U. S. Army, in charge of the Medical and Microscopical Sections of the Museum.

Lectures on the Diseases of Women. By Charles West, M. D., Fellow of the Royal College of Physicians; Examiner of Midwifery at the University of London, etc., etc. Thlrd American from the third and revised London edition. Philadelphia: Henry C. Lea, 1867. For sale by Theodore Butler.

A Practical Treatise on Shock after Surgical Operations and Injuries; with special reference to shock caused by railway accidents. By Edwin Morris, M. D., F. R. S., Surgeon to the Spalding Dispensary and Union Infirmary. Philadelphia: J. B. Lippincott & Co., 1868. For sale by Breed, Lent & Co.

Headaches; their Causes and their Cure. By Henry G. Wright, M. D., M. R. C. S. L., L. S. A., Member of the Royal College of Physicians of England, etc. From the fourth London edition. Philadelphia: Lindsay & Blakiston, 1867. For sale by Theodore Butler.

A Treatise on the Cause of Exhausted Vitality; or Abuses of the Sexual Function. By E. P. Miller, M. D., Physician to the Hygiene Institute and Turkish Baths. New York, 1867.

The Physician's Hand Book for 1868. By William Elmer, M. D. New York: W. A. Townsend & Adams, publishers, 434 Broome street. For sale by Theodore Butler.

Transactions of the Medical Society of the State of Pennsylvania, at its Eighteenth Annual Session, held at Pittsburgh, June, 1867. Fourth series, part 3d, published by the Society.

Report of the Proceedings of the Association of Medical Superintendents of American Iastitutions for the Insane, 1867.

New Researches on the Therapeutic Use of Manganese as an adjuvant of Iron. By Dr. J. E. Pétrequin, late Head Surgeon of the Hotel Dieu of Lyons, etc.

Superintendent's Report of the New York Institute for the Blind at Binghamton, transmitted to the Legislature February 9, 1867.

Forty-second Annual Report of the Massachusetts Charitable Eye and Ear Infirmary, for the year ending September 30, 1867.

Conveyance of Cholera from Ireland to Canada and the United States Indian Territory, in 1832. By John C. Peters, M. D., of New York.

Base of Brain with Nerves emerging. Arranged by S. W. Wetmore, M. D., Demonstrator of Anatomy in the Medical Department of the University of Buffalo, N. Y.

A Complete List of the Muscles of the Human Body. By William Little, M. D., Chicago, Ill.

Smallpox Vaccination. By R. M. Lakey, M. D., Chicago, Illinois.

